# Dual-route embedding-aware graph neural networks for drug repositioning

**DOI:** 10.1093/bib/bbaf555

**Published:** 2025-10-27

**Authors:** Yanlong Zhao, Yixiao Chen, Jiawen Du, Jun Wen, Quan Sun, Ren Wang, Can Chen

**Affiliations:** Department of Electrical and Computer Engineering, University of Rochester, 120 Trustee Road, Rochester, NY 14620, United States; Department of Computer Science, University of North Carolina at Chapel Hill, 201 S Columbia Street, Chapel Hill, NC 27599, United States; Department of Biostatistics, University of North Carolina at Chapel Hill, 135 Dauer Drive, Chapel Hill, NC, 27599, United States; Department of Biomedical Informatics, Harvard Medical School, Harvard University, 10 Shattuck Street, Boston, MA 02115, United States; Center for Computational and Genomic Medicine, Children’s Hospital of Philadelphia, 3401 Civic Center Boulevard, Philadelphia, PA 19104, United States; Department of Pathology and Laboratory Medicine, University of Pennsylvania Perelman School of Medicine, 3400 Spruce Street, Philadelphia, PA 19104, United States; Department of Electrical and Computer Engineering, Illinois Institute of Technology, 3301 S Dearborn Street, Chicago, IL 60616, United States; Department of Biostatistics, University of North Carolina at Chapel Hill, 135 Dauer Drive, Chapel Hill, NC, 27599, United States; School of Data Science and Society, University of North Carolina at Chapel Hill, 211 Manning Drive, Chapel Hill, NC 27599, United States; Department of Mathematics, University of North Carolina at Chapel Hill, 120 E Cameron Avenue, Chapel Hill, NC 27599, United States; Carolina Health Informatics Program, University of North Carolina at Chapel Hill, 335 S Columbia Street, Chapel Hill, NC 27599, United States

**Keywords:** drug repositioning, graph neural networks, multiview learning, biomedical language models

## Abstract

Drug repositioning presents a compelling strategy to accelerate therapeutic development by uncovering new indications for existing compounds. However, current computational methods are often limited in their ability to integrate heterogeneous biomedical data and model the intricate, multiscale relationships underlying drug–disease associations, while large-scale experimental validation remains prohibitively resource-intensive. Here, we present DREAM-GNN (Dual-Route Embedding-Aware Model for Graph Neural Networks), a multiview deep graph learning framework that incorporates biomedical domain knowledge with two complementary graphs capturing both topological structure and feature similarity to enable accurate and biologically meaningful prediction of drug–disease associations. Extensive experiments on benchmark datasets demonstrate that DREAM-GNN significantly outperforms current state-of-the-art methods in recovering artificially removed repositioning candidates, including in scenarios involving unseen drugs and diseases. These results establish DREAM-GNN as a robust and generalizable computational framework with broad potential to streamline drug discovery and advance precision medicine.

## Introduction

Drug repositioning, also known as drug repurposing, is the process of identifying new therapeutic uses for existing or previously failed drugs [1–6]. In contrast to traditional drug discovery, which is time-consuming, costly, and associated with a high failure rate, drug repositioning leverages established pharmacological and safety profiles to significantly reduce development time and expense required to bring treatments to market [7–9]. This paradigm has gained increasing attention in recent years as a valuable strategy for addressing unmet medical needs, particularly in complex or rare diseases where conventional discovery pipelines face limitations [10–13]. Notable successes, such as the repositioning of sildenafil, initially developed for angina and later approved for erectile dysfunction and pulmonary hypertension, highlight the clinical and commercial potential of this approach [14–16]. Recent applications extend to oncology, drug resistance mitigation, and personalized therapeutics, further reinforcing the promise of repositioning to diversify and modernize treatment landscapes [17–21]. Therefore, scalable, accurate, and data-driven methods are essential for drug repositioning, as large-scale experimental validation of candidate drug–disease associations remains prohibitively resource-intensive.

Computational methods have become central to modern repositioning efforts, enabling systematic prioritization of candidate associations across vast biomedical datasets. Early efforts predominantly relied on matrix factorization techniques, which project observed drug–disease interactions into a low-rank latent space while incorporating biological priors through drug and disease similarity matrices. Representative examples include drug repositioning recommendation system (DRRS) [22], which applies singular value thresholding to integrate disease–disease, drug–drug, and drug–disease relationships, and bounded nuclear norm regularization (BNNR) [23], which uses bounded nuclear norm regularization to fuse multiple biological similarity measures. Other variants, such as iDrug [24] and similarity constrained matrix factorization method for the drug-disease association prediction (SCMFDD) [25], exploit cross-network structures and semantic similarity constraints to improve biological interpretability. More recent extensions incorporate multiview learning or genomic topology to enhance predictive performance [26, 27]. Yet, matrix factorization-based methods struggle to capture complex, nonlinear drug–disease interactions and become computationally demanding on large heterogeneous datasets.

The rapid proliferation of high-throughput biomedical data has fueled growing interest in machine learning approaches for drug repositioning [28–32]. These methods typically frame repositioning as a supervised learning task, predicting drug–disease associations from structured features derived from molecular, phenotypic, or network data. Pioneering models include PREDICT [33], which integrates drug–drug and disease–disease similarity measures within a logistic regression framework to infer associations, alongside other methods that employ classifiers such as random forests to predict drug–target interactions [34]. These efforts laid the groundwork for more sophisticated deep learning models. For example, deepDR [35] employs multimodal autoencoders to learn high-level feature representations from heterogeneous drug–disease networks; CBPred [36] leverages multipath similarity to improve predictive accuracy; and dual-feature drug repurposing neural network (DFDRNN) [37] introduces a deep fusion network that integrates diverse biological data sources through hierarchical deep learning modules. Despite these advances, such machine learning approaches generally treat neighboring nodes as independent, limiting their ability to model the complex relational patterns that underlie pharmacological and pathological processes.

Graph neural networks (GNNs) have recently emerged as leading approaches for drug repositioning by explicitly modeling both node features and network topology. Initial efforts focus on capturing local structural information and separately integrating similarity networks [38], while more advanced architectures incorporate attention mechanisms and heterogeneous information fusion, exemplified by models such as layer attention graph convolutional network (LAGCN) [39] and heterogeneous information fusion graph convolutional network (DRHGCN) [40]. Subsequent methods like drug repositioning approach based on weighted bilinear neural collaborative filtering (DRWBNCF) [41] and partner-specific drug repositioning approach based on graph convolutional network (PSGCN) [42] improve the modeling of context-specific dependencies through weighted convolution operations and subgraph-based classification strategies. The latest state-of-the-art frameworks, including weighted local information augmented graph neural network model (DRAGNN) [43] and adaptive graph convolutional network approach for drug repositioning (AdaDR) [44], incorporate weighted aggregation schemes and adaptive graph convolutions to more effectively capture the complexity of biological interactions and improve model robustness. However, these GNN-based approaches still face significant challenges in effectively integrating heterogeneous multiview data. More critically, they often fall short of fully leveraging available domain knowledge, such as drug chemical structures and disease semantics, limiting their biological interpretability and predictive power. These highlight a pressing need for novel GNN-based frameworks that can seamlessly integrate multiview data and embed rich biochemical knowledge to accurately predict drug–disease associations.

To overcome these limitations, we introduce DREAM-GNN (Dual-Route Embedding-Aware Model for Graph Neural Networks), a novel framework that integrates multimodal, pretrained embeddings of drugs and diseases with two complementary graph encoders, consisting of one modeling network topology and the other capturing feature similarity, to enable accurate and biologically meaningful prediction of drug–disease associations. Through comprehensive evaluations across three benchmark datasets (Gdataset [33], Cdataset [45], and LRSSL [46]), DREAM-GNN consistently outperforms state-of-the-art methods in identifying artificially removed drug repositioning candidates, even in scenarios with unseen drugs and diseases. These results demonstrate that DREAM-GNN delivers a biologically grounded and highly effective solution for computational drug repositioning.

## Materials and methods

### Overview of DREAM-GNN

**Figure 1:**
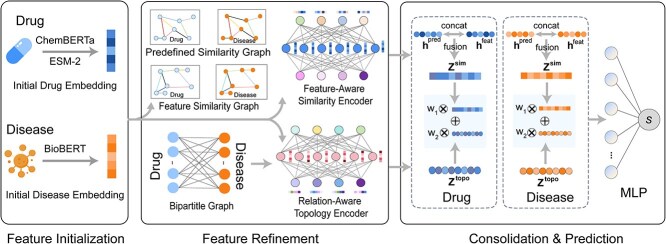
DREAM-GNN workflow: drug embeddings are generated by encoding chemical SMILES with ChemBERTa and protein sequences with ESM-2, while disease embeddings are obtained using BioBERT on curated textual definitions; two types of graphs are constructed, namely similarity graphs (domain-defined and embedding-derived $k$-nearest neighbor similarity) and a bipartite drug–disease interaction graph with a feature-aware similarity encoder applying GNNs separately to the drug and disease similarity graphs and a relation-aware topology encoder refining node embeddings over the bipartite interaction graph; similarity-based and topology-informed embeddings are fused using a nonlinear layer and attention mechanism, then decoded with an MLP to predict drug–disease associations (notations: $h^{\mathrm{pred}}$ and $h^{\mathrm{feat}}$ denote the node representations learned by GNNs on the predefined similarity graph and the embedding-derived KNN graph, respectively, $z^{\mathrm{sim}}$ denotes the unified similarity embedding obtained by fusing $[h^{\mathrm{pred}};h^{\mathrm{feat}}]$  $z^{\mathrm{topo}}$ denotes the topology-route embedding from the relation-aware encoder on the bipartite graph, and $w_{1}$ and $w_{2}$ are the attention weights for topology and similarity views; operators: “concat” denotes vector concatenation, $\otimes $ denotes scalar-vector scaling, and $\oplus $ denotes vector addition).

We frame drug repositioning as heterogeneous link prediction on a bipartite drug–disease graph. DREAM-GNN first generates initial drug and disease embeddings by leveraging pretrained large language-based models: ChemBERTa [47], ESM-2 [48], and BioBERT [49]. These embeddings are then refined in parallel through a relation-aware topology encoder and a feature-aware similarity encoder. Finally, an additive-attention gate integrates the dual views into a unified embedding, which is passed through a multilayer perceptron to generate predicted scores for drug–disease pairs. The complete workflow of DREAM-GNN is illustrated in Fig. 1.

### Feature initialization

Effective feature initialization is critical for capturing meaningful biological semantics and enabling accurate downstream prediction in graph-based drug repositioning models. To represent the diverse properties of drugs and diseases, DREAM-GNN employs modality-specific embedding strategies based on pretrained large language models tailored to each entity type, including small-molecule drugs, protein therapeutics, and diseases. These heterogeneous embeddings are then harmonized into a unified feature space, ensuring compatibility and facilitating seamless integration within the GNN architecture.

#### Drug initial embeddings

For small-molecule drugs, embeddings are derived from canonical SMILES (Simplified Molecular Input Line Entry System) strings using ChemBERTa [47], a transformer model pretrained on extensive chemical corpora. SMILES is a chemical notation system that represents molecular structures as compact ASCII text strings, encoding atoms, bonds, branching, and stereochemistry in a linear format, i.e. particularly well suited for computational processing and machine learning applications [50]. The canonical form ensures a unique, standardized representation for each molecule, eliminating structural redundancy and enabling consistent molecular encoding. Each canonical SMILES string is tokenized via byte-pair encoding, then truncated or padded to a fixed length of 510 tokens. These sequences are processed by ChemBERTa to generate contextual hidden states, which are pooled using masked mean pooling over nonpadding tokens to produce a 1024-dimensional embedding. For protein therapeutics, a similar strategy is applied using amino acid sequences and ESM-2 [48], a 33-layer transformer protein language model. Sequences are augmented with beginning- and end-of-sequence tokens and fed into ESM-2, producing 1280-dimensional hidden states per token. Averaging the amino acid token states, excluding the boundary tokens, yields a 1280-dimensional embedding that captures the protein’s global semantic representation without positional bias.

To unify small-molecule and protein embeddings into a common feature space, the 1024-dimensional drug embeddings are zero-padded to match the 1280-dimensional protein vectors. These harmonized embeddings are then concatenated into a single feature matrix of size $N_{d} \times 1280$, where each row corresponds to a drug, whether small molecule or protein therapeutic. To reduce redundancy and compress this unified matrix, principal component analysis is applied. Following common practice in dimensionality reduction, we select the top 768 components to balance computational efficiency with information retention, yielding a compact $N_{d} \times 768$ matrix that serves as the final drug embeddings for downstream graph-based inference. The choice of 768 is determined via a parameter grid search.

#### Disease initial embeddings

Each disease is encoded by converting its curated free-text description into a fixed-length embedding. Textual definitions from OMIM [51] and MeSH [52] are tokenized and input into the pretrained BioBERT language model [49], which generates a 768-dimensional contextual vector for each token, capturing nuanced biomedical semantics. A single representation per description is obtained by applying masked mean pooling to average only the vectors of substantive (nonpadding) tokens, resulting in a 768-dimensional embedding for each disease. Repeating this process for all $N_{m}$ diseases produces a disease feature matrix with domain-aware embeddings ready for downstream analysis.

### Feature refinement

DREAM-GNN refines initial drug and disease embeddings through a dual-route GNN that jointly captures two complementary sources of information: the explicit topology of known interactions and the implicit structure encoded in feature similarities.

#### Relation-aware topology encoder

The relation-aware topology encoder operates on a bipartite drug–disease graph that incorporates two distinct edge types to distinguish evidence: $\mathcal{R}_{\text{known}}$ represents clinically validated therapeutic associations, while $\mathcal{R}_{\text{unknown}}$ denotes pairs with no known interaction, sampled as negative examples. A multilayer GNN based on the graph convolutional matrix completion operator [53] is employed to encode the relational topology. At each layer $l$, a node (drug or disease) embedding $\mathbf{h}_{v}^{(l)}$ is refined by aggregating normalized messages from its neighbors under each relation type:


(1) \begin{align*}& \mathbf{h}_{v}^{(l+1)} = \sigma\left( \sum_{r \in \mathcal{R}} \sum_{u \in \mathcal{N}_{r}(v)} \frac{1}{\sqrt{|\mathcal{N}_{r}(u)|\, |\mathcal{N}_{r}(v)|}} \mathbf{W}^{(l)}_{r} \mathbf{h}^{(l)}_{u} \right),\end{align*}

where $\mathcal{N}_{r}(v)$ denotes the set of neighbors of node $v$ under relation $r$, and $\sigma $ is a nonlinear activation function. To balance expressiveness and parameter efficiency, each relation-specific weight matrix $\mathbf{W}^{(l)}_{r}$ is factorized via basis decomposition: $\mathbf{W}^{(l)}_{r} = \sum _{b=1}^{B} a_{rb}^{(l)} \mathbf{V}_{b}^{(l)}$, where $\mathbf{V}_{b}^{(l)}$ are shared basis matrices and $a_{rb}^{(l)}$ are relation-specific coefficients. The final topology-aware embedding $\mathbf{z}_{v}^{\text{topo}}$ is obtained by averaging the node representations across all $L$ layers: $\mathbf{z}_{v}^{\text{topo}} = \frac{1}{L} \sum _{l=1}^{L} \mathbf{h}_{v}^{(l)}$, resulting in a multiscale encoding that integrates signals from different neighborhood depths. This relation-aware topology encoder enables the model to distinguish between interaction types and effectively capture the structural heterogeneity of the drug–disease network.

#### Feature-aware similarity encoder

The feature-aware similarity encoder is designed to capture rich, multigranular similarity patterns that complement the explicit relational topology. For both drugs and diseases, two distinct $k$-nearest neighbor similarity graphs are constructed: (i) a predefined similarity graph, with drug, protein, and disease similarities obtained from published datasets [43, 44], where drug similarities are based on Tanimoto coefficients from chemical fingerprints and disease similarities on semantic scores derived from medical ontologies; and (ii) a feature-based similarity graph, which encodes latent relationships by computing cosine similarity over the initial embeddings. Each of the four similarity graphs (two for drugs, two for diseases) is processed using a two-layer graph convolutional network without binarization. At each layer $l$, the node embeddings $\mathbf{H}^{(l)}$ are updated according to the propagation rule:


(2) \begin{align*}& \mathbf{H}^{(l+1)} = \sigma(\hat{\mathbf{A}}\mathbf{H}^{(l)}\mathbf{W}^{(l)} + \mathbf{b}^{(l)}),\end{align*}

where $\hat{\mathbf{A}} = \mathbf{D}^{-\frac{1}{2}}(\mathbf{A} + \mathbf{I})\mathbf{D}^{-\frac{1}{2}}$ is the symmetrically normalized adjacency matrix with self-loops. For each entity type (drug or disease), the representations learned from the predefined similarity graph ($\mathbf{h}_{v}^{\text{pred}}$) and the feature-derived graph ($\mathbf{h}_{v}^{\text{feat}}$) are fused to form a unified similarity embedding:


(3) \begin{align*}& \mathbf{z}_{v}^{\text{sim}} = \text{Dropout}\left(\text{ReLU}\left(\mathbf{W}_{\text{fuse}} [\mathbf{h}_{v}^{\text{pred}}; \text{} \mathbf{h}_{v}^{\text{feat}}] + \mathbf{b}_{\text{fuse}}\right)\right).\end{align*}

This feature-aware similarity encoder effectively captures both latent and predefined similarity patterns, enhancing the model’s capacity to represent complex and nuanced relationships within drug and disease entities.

### Consolidation and prediction

To obtain a unified representation for each drug and disease, the topology-aware embedding ($\mathbf{z}_{v}^{\text{topo}}$) and similarity-aware embedding ($\mathbf{z}_{v}^{\text{sim}}$) are combined using a learnable attention mechanism. The two embeddings are first stacked as $\mathbf{Z}_{v} = [\mathbf{z}_{v}^{\text{topo}}; \mathbf{z}_{v}^{\text{sim}}] \in \mathbb{R}^{2 \times d}$. An attention network computes a normalized weight vector $\mathbf{w}=[w_{1} \text{} w_{2}] \in \mathbb{R}^{2}$ that reflects the relative importance of each view:


(4) \begin{align*}& \mathbf{w} = \text{Softmax}\left(\mathbf{W}_{2} \tanh(\mathbf{W}_{1} \mathbf{Z}_{v})\right),\end{align*}

where $\mathbf{W}_{1}$ and $\mathbf{W}_{2}$ are learnable weight matrices. The final fused embedding is computed as a weighted sum of the two components:


(5) \begin{align*}& \mathbf{z}_{v} = \text{Dropout}(w_{1} \mathbf{z}_{v}^{\text{topo}} + w_{2} \mathbf{z}_{v}^{\text{sim}}).\end{align*}

This attention-based fusion enables the model to adaptively integrate complementary structural and similarity information, yielding more informative and discriminative representations for both drugs and diseases. Finally, the fused embeddings of a drug $v_{i}$ (denoted by $\mathbf{z}_{i}$) and a disease $v_{j}$ (denoted by $\mathbf{z}_{j}$) are concatenated and passed through a three-layer multilayer perceptron (MLP) decoder, which outputs a single predicted score $s_{ij}=\text{Sigmoid}(\text{MLP}([\mathbf{z}_{i}; \text{} \mathbf{z}_{j}]))$. The model is trained end-to-end by minimizing the binary cross-entropy loss between the predicted scores and ground-truth labels, ensuring numerical stability and effective optimization.

### Prediction for unseen drugs and diseases

We address the challenge of strict cold-start prediction for drugs and diseases that are absent from the training graph, a common scenario as novel compounds and understudied indications continue to emerge. Building on prior work in graph model generalization [54, 55], our approach leverages similarity information derived from pretrained embeddings: ChemBERTa [47] for small molecules, ESM-2 [48] for proteins/targets, and BioBERT [49] for diseases. Inference proceeds in three stages. First, for each unseen entity, we identify its top-k most similar training entities in the original embedding space using cosine similarity, ensuring independence from learned association patterns. Second, we aggregate the trained representations of these neighbors to form a pseudo-query for the unseen entity using temperature-scaled attention, where attention weights are derived from cosine similarities in the original embedding space and applied to neighbors’ trained embeddings. Finally, the pseudo-query is scored against candidate partners by a fixed decoder whose parameters remain unchanged from training. This approach enables DREAM-GNN to generate accurate and reliable predictions for novel drugs and diseases.

### Advantages over existing methods

While rooted in the foundational design of GNNs, DREAM-GNN introduces targeted architectural and representational innovations that address the unique challenges of drug–disease association prediction. These advances enable it to surpass existing state-of-the-art methods in both accuracy and generalizability. First, DREAM-GNN emphasizes the integration of rich, multimodal features derived from diverse sources (SMILES, protein sequences, anddisease texts) using specialized pretrained models to create informative initial node embeddings. This contrasts with many prior methods that rely on simplistic node features such as one-hot encoding or random initialization. Second, DREAM-GNN adopts a dual-route architecture: a relation-aware topology encoder captures structural information from known drug–disease associations, while a feature-aware similarity encoder extracts relational patterns from similarity graphs constructed using both raw descriptors and learned embeddings. This design enables the model to fuse complementary insights from interaction topology and feature space geometry, an advantage over conventional single-graph approaches that often overlook such synergy. Furthermore, DREAM-GNN incorporates several robust learning strategies, including attention dropout, basis decomposition of weight matrices, and data augmentation via Gaussian feature perturbation ($\sigma =0.05$) and edge dropout ($P=.10$), to enhance generalization and reduce the risk of overfitting.

## Results

### Datasets

To evaluate the performance of our proposed model, we utilize three widely used benchmark datasets: Gdataset [33], Cdataset [45], and LRSSL [46]. These datasets contain validated drug–disease associations and have been commonly adopted in drug repositioning studies.

- Gdataset [33], often regarded as the gold-standard benchmark, contains 1933 experimentally validated drug–disease pairs linking 593 small-molecule drugs from DrugBank to 313 Mendelian diseases cataloged in OMIM [56]. Its rigorous manual curation and balanced coverage of both drug and disease spaces have established it as a *de facto* standard for evaluating predictive models.
- Cdataset [45], introduced by Luo *et al*., expands the evaluation scope to 2352 drug–disease associations spanning 663 drugs and 409 diseases. Compared to Gdataset, it presents a denser interaction matrix and a slightly broader chemical space, allowing for a complementary assessment of model performance under more heterogeneous and noisier real-world conditions.
- The LRSSL dataset [46] expands interaction coverage to 3051 experimentally verified associations involving 763 FDA-approved drugs and 681 diseases. In addition to the bipartite drug–disease network, it includes three drug–drug similarity matrices based on chemical substructure, target protein domains, and Gene Ontology term overlap, as well as a semantic disease–disease similarity matrix derived from OMIM [56], enabling methods that incorporate auxiliary similarity information.

### Baselines

To demonstrate the effectiveness of our proposed model, we compare its performance against several state-of-the-art methods for drug repositioning. These baseline methods represent a range of techniques, including matrix factorization and GNN-based approaches.

- BNNR [23] formulates drug repositioning as a matrix completion problem. It utilizes bounded nuclear norm regularization to predict potential drug–disease associations by recovering the underlying low-rank structure of the association matrix.
- DRAGNN [43] is a recent GNN-based approach that improves drug repositioning predictions by integrating weighted local neighborhood information. It exploits the inherent graph structure of drug–disease interactions and assigns importance to neighboring nodes during message aggregation, enhancing the relevance of learned representations.
- AdaDR [44] introduces an adaptive graph convolutional framework for drug repositioning, where the model dynamically learns or adjusts graph structures to better reflect the underlying topology of drug–disease interactions. This adaptability enables more effective information propagation tailored to the specific characteristics of the network.
- DFDRNN [37] is a recent deep learning framework for drug–disease association prediction that introduces a deep fusion neural network to integrate diverse biological data sources. By combining heterogeneous information, the model aims to enhance predictive accuracy and uncover more reliable repositioning candidates.

### Experiment setup

#### Evaluation protocol

To rigorously assess the performance of our model and baseline methods, we adopt a 10-fold cross-validation strategy across all datasets. For each dataset, the set of known drug–disease associations (positive samples) and the complete set of unknown pairs (negative samples) are partitioned into 10 mutually exclusive folds. Unlike methods that employ downsampling strategies to balance class distributions, we utilize all available negative samples without subsampling, thus evaluating our model’s performance on the full scope of the drug–disease interaction space. This comprehensive approach more accurately reflects real-world scenarios, where most drugs and diseases are known but many associations remain unobserved. In each cross-validation round, one-fold is held out as the test set, while the remaining nine-folds are used for training. Both positive and negative samples are split independently to ensure proper stratification. This process is repeated 10 times, ensuring that each fold is used exactly once for evaluation. Performance metrics are averaged across the folds to yield a robust estimate of generalization. To avoid information leakage, we rebuild the training graph using only training edges and recompute fold-specific similarities based solely on training nodes without using any label information from test entities for each cross-validation fold.

#### Evaluation metrics

We assess the predictive performance using two standard metrics widely adopted in binary classification and drug repositioning tasks: the area under the receiver operating characteristic curve (AUROC) and the area under the precision-recall curve (AUPRC). AUROC evaluates the model’s ability to distinguish between positive and negative associations across various thresholds, while AUPRC is particularly informative for imbalanced datasets, measuring the trade-off between precision and recall. Higher values for both AUROC and AUPRC indicate better prediction performance.

#### Hyperparameters

We perform a grid search to identify optimal hyperparameter configurations using the LRSSL dataset. This strategic choice is motivated by two key considerations: (i) LRSSL is the largest and most comprehensive benchmark among the three datasets, containing 3051 drug–disease associations across 763 drugs and 681 diseases, providing sufficient data diversity for robust parameter selection; (ii) Hyperparameters optimized on this more complex and heterogeneous dataset are more likely to generalize well to the smaller and simpler Gdataset and Cdataset, following the principle that models tuned on challenging tasks typically transfer effectively to easier ones. Hyperparameter settings that yield the highest average AUPRC across cross-validation folds are selected for final evaluation. Table 1 summarizes the explored search space for each hyperparameter, with the optimal values (in bold) corresponding to those that achieve the best performance during training and validation on LRSSL. The identified optimal hyperparameters from LRSSL are then directly applied to Gdataset and Cdataset without further tuning, ensuring a fair comparison and demonstrating the generalizability of our approach.

**Table 1:** Hyperparameter selection for DREAM-GNN.

Component	Hyperparameter	Search space
Initial embedding	Drug dimension	{512, **768**, 1280}
Topology encoder	Number of layers	{2, **3**, 4}
	Aggregation units	{768, **1024**, 1536, 2048}
	Output units	{96, **128**, 256}
	Basis decomposition ($B$)	{2, **4**, 8}
Similarity encoder	Hidden units (layer 1)	{512, **768**, 1024}
	Hidden units (layer 2)	{96, **128**, 256}
	$k$ -NN neighbors ($k$)	{2, **4**, 8, 16}
Regularization	Dropout rate	{0.1, 0.2, **0.3**, 0.4}
	Attention dropout	{0.0, **0.1**, 0.2}
Optimization	Learning rate	{1e-4, 1e-3, **2e-3**, 5e-3, 1e-2}
	Weight decay	{1e-6, **1e-5**, 1e-4}

Grid search is performed on the LRSSL dataset, with optimal values shown in bold.

### Performance comparison and analysis

**Figure 2:**
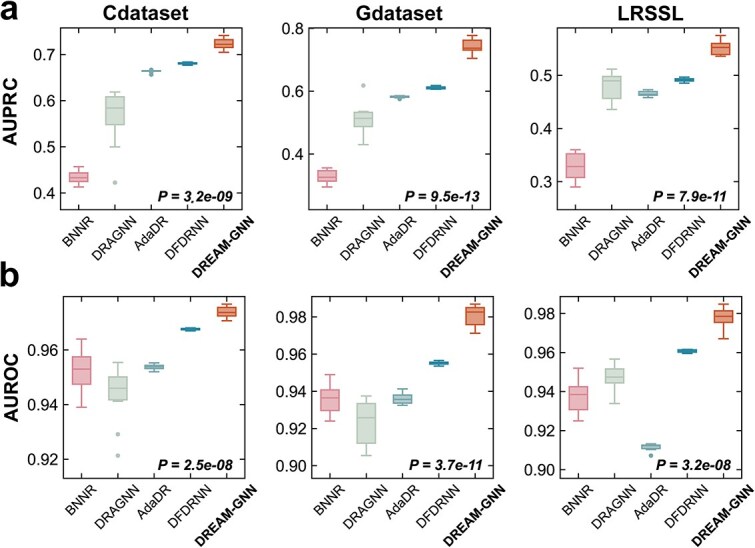
Comparison of DREAM-GNN with existing methods across three datasets: DREAM-GNN outperforms BNNR, DRAGNN, AdaDR, and DFDRNN on both AUPRC (a) and AUROC (b) metrics across Cdataset, Gdataset, and LRSSL (boxplots reflect performance distributions over 10-fold cross-validation, and $P$-values indicate statistical significance from paired $t$-tests against the strongest baseline for each dataset).

DREAM-GNN significantly outperforms existing state-of-the-art methods across all the three benchmark datasets, demonstrating both strong predictive accuracy and stability under varying data conditions (Fig. 2). On the widely used Cdataset, DREAM-GNN achieves median scores of 72.20% AUPRC and 97.38% AUROC, surpassing the second-best method DFDRNN by 4.13 percentage points in AUPRC ($P$-value = 3.2E-9, paired $t$-test) and 0.61 points in AUROC ($P$-value = 2.5E-8). Other baselines including AdaDR, DRAGNN, and BNNR lag considerably behind with poor predictive power. Performance gains are even more striking on the more heterogeneous Gdataset, where DREAM-GNN achieves 73.72% AUPRC and 98.27% AUROC. In comparison, DFDRNN trails by 12.73 points in AUPRC ($P$-value = 9.5E-13) and 2.77 points in AUROC ($P$-value = 3.7E-11), indicating DREAM-GNN’s capacity to generalize effectively across noisier and more complex interactions. Evaluation on the LRSSL dataset further underscores DREAM-GNN’s robustness. It reaches 55.24% AUPRC and 97.86% AUROC, outperforming DFDRNN by 6.11 points in AUPRC ($P$-value = 7.9E-11) and 1.76 points in AUROC ($P$-value = 3.2E-8). Additional performance comparisons using F1-score and recall are reported in Supplementary data.

Notably, DREAM-GNN delivers substantial and consistent gains in AUPRC across all evaluated datasets, underscoring its effectiveness in settings characterized by severe class imbalance, a common hallmark of drug-disease association tasks. Unlike AUROC, AUPRC is more sensitive to the performance on the minority (positive) class and thus better reflects real-world utility in identifying novel therapeutic associations. On the challenging Gdataset, e.g. DREAM-GNN achieves an AUPRC of 73.72%, representing a 12.73-point improvement over the next-best method DFDRNN. Similar trends are observed on Cdataset and LRSSL, with DREAM-GNN outperforming all baselines by wide and statistically significant margins. Furthermore, DREAM-GNN exhibits significantly lower variability compared to methods like BNNR and DRAGNN, indicating superior generalizability and stability. These improvements suggest that the model effectively prioritizes biologically meaningful associations amidst overwhelming noise, crucial for practical drug repositioning efforts.

### Validation with unseen drugs and diseases

**Figure 3:**
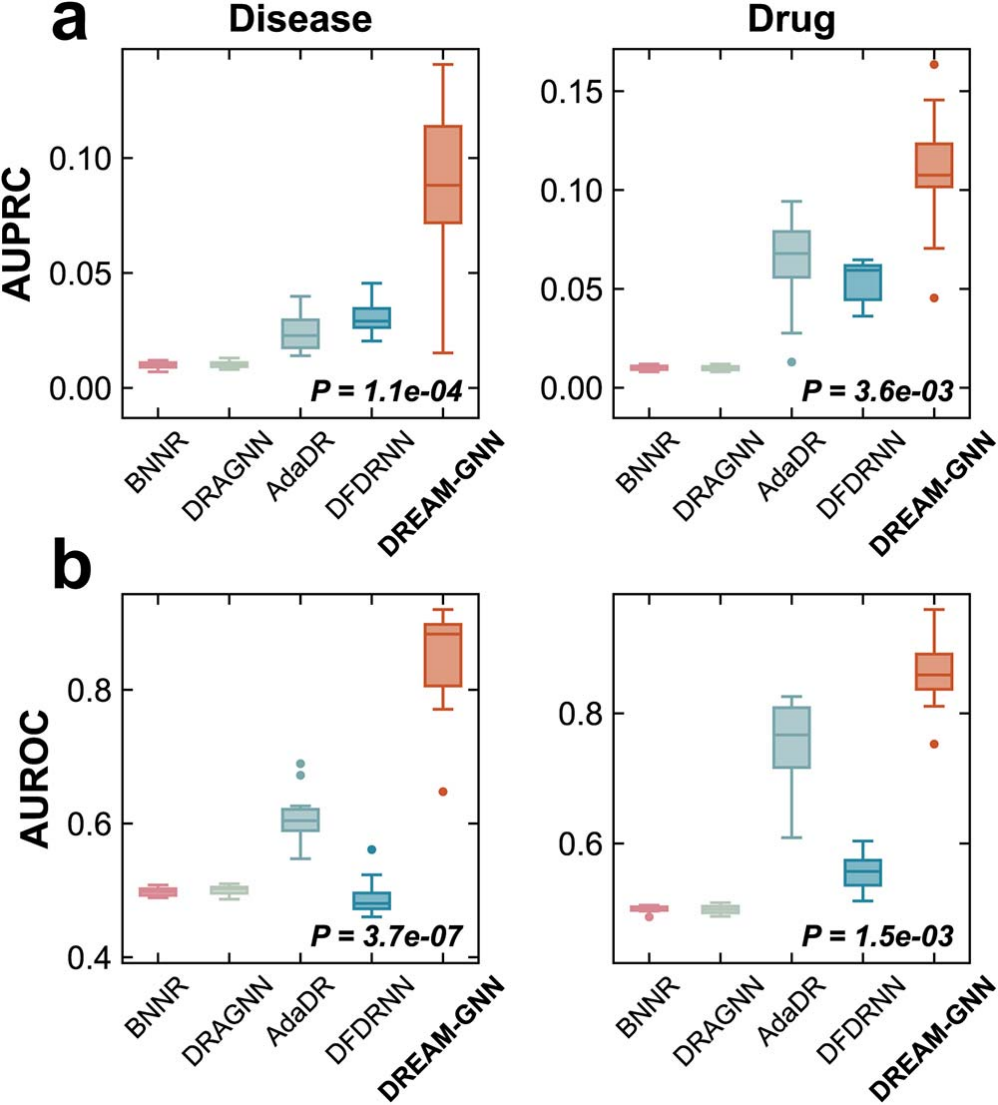
Comparison of DREAM-GNN with existing methods across on the Gdataset dataset for unseen disease and drug prediction: DREAM-GNN outperforms BNNR, DRAGNN, AdaDR, and DFDRNN on both AUPRC (a) and AUROC (b) metrics (boxplots reflect performance distributions over 10-fold cross-validation, and $P$-values indicate statistical significance from paired $t$-tests against the strongest baseline for each dataset).

To assess strict inductive generalization beyond the training graph, we evaluate two settings, unseen diseases and unseen drugs, under 10-fold cross-validation using the Gdataset dataset. In each fold, all held-out diseases or drugs are removed from the training graphs and excluded from any fold-specific similarity construction. Cold-start inference uses the trained model without any retraining or graph rebuilding. As shown in Fig. 3, DREAM-GNN attains the highest AUPRC and AUROC in both settings. In the unseen disease setting (left panels), DREAM-GNN’s AUPRC distribution is markedly shifted upward relative to BNNR, DRAGNN, AdaDR, and DFDRNN, and the AUROC shows a similar separation; both gains are statistically significant (AUPRC: $P$-value = 1.1E-4; AUROC: $P$-value = 3.7E-7; paired $t$-test). In the unseen drug setting (right panels), DREAM-GNN again leads by a large margin, with significant improvements over the second-best baseline AdaDR for both AUPRC ($P$-value = 3.6E-3) and AUROC ($P$-value = 1.5E-3). These cold-start outcomes demonstrate that DREAM-GNN not only fits known graphs well but also generalizes to truly novel entities, a critical requirement for practical drug repositioning where new compounds and understudied indications frequently arise.

### Ablation study

To systematically investigate the effectiveness and contributions of each component within the DREAM-GNN framework, we conduct a comprehensive ablation study on the LRSSL dataset (Table 2). Specifically, we examine the following four critical variants: (i) DREAM-GNN-w/o-attention-dropout, which removes the dropout regularization from the attention fusion mechanism; (ii) DREAM-GNN-w/o-parameter-sharing, where parameters in the graph convolutional layers are independently learned without sharing; (iii) DREAM-GNN-w/o-augmentation, omitting all data augmentation strategies, including Gaussian feature noise and edge dropout; (iv) DREAM-GNN-w/o-node-feature-embedding, which replaces the pretrained embeddings derived from ChemBERTa [47], ESM-2 [48], and BioBERT [49] with random initialization; (v) DREAM-GNN-topology-only which removes the similarity encoder and all cross-route fusion; and (vi) DREAM-GNN-similarity-only which removes the topology encoder and operate solely on similarity graphs/embeddings.

**Table 2:** Ablation study results for DREAM-GNN: average test AUROC and AUPRC are reported with standard deviation over multiple runs.

Model/strategy	AUROC	AUPRC
DREAM-GNN	0.984 $\pm $ 0.005	0.575 $\pm $ 0.057
w/o Attention dropout	0.980 $\pm $ 0.004	0.564 $\pm $ 0.048
w/o Parameter sharing	0.976 $\pm $ 0.005	0.355 $\pm $ 0.149
w/o Augmentation	0.972 $\pm $ 0.025	0.541 $\pm $ 0.088
w/o Node feature embedding	0.913 $\pm $ 0.045	0.463 $\pm $ 0.012
Topology only	0.982 $\pm $ 0.007	0.305 $\pm $ 0.089
Similarity only	0.925 $\pm $ 0.007	0.444 $\pm $ 0.041

The results clearly illustrate that each component plays an essential role in the overall predictive performance of DREAM-GNN. Removing pre-trained node embeddings leads to the most pronounced performance drop, substantially decreasing both AUPRC and AUROC, indicating the critical importance of incorporating rich, domain-specific feature information for capturing intrinsic biological semantics. Similarly, disabling parameter sharing markedly deteriorates AUPRC, underscoring the benefits of parameter efficiency and improved generalization achieved through shared convolutional weights. The absence of data augmentation strategies, such as feature noise and edge dropout, also negatively impacts model robustness, suggesting these augmentations effectively mitigate overfitting. Removing dropout in the attention fusion mechanism leads to a moderate yet noticeable performance reduction, highlighting its role in enhancing the stability and generalization capabilities of the learned attention weights. Finally, the single-route ablations reveal that topology-only favors AUROC and similarity-only favors AUPRC, whereas the dual-route DREAM-GNN improves both metrics simultaneously. Collectively, these findings confirm that each proposed component contributes meaningfully to its overall predictive accuracy, robustness, and generalization capability.

## Discussion

In this work, we present DREAM-GNN framework tailored to tackle the challenges of data heterogeneity in computational drug repositioning. DREAM-GNN systematically integrates rich, modality-specific embeddings derived from state-of-the-art pretrained language models, including ChemBERTa, ESM-2, and BioBERT, to establish a comprehensive initial representation of drugs and diseases. Its dual-encoder architecture jointly models explicit interaction topologies and implicit semantic similarities, while a learnable attention mechanism dynamically fuses these complementary views. This design enables DREAM-GNN to learn robust and discriminative representations for accurate prediction of novel drug–disease associations. Extensive evaluation across three large-scale benchmark datasets demonstrates that DREAM-GNN consistently outperforms existing methods, highlighting its effectiveness and generalizability.

Beyond aggregate metrics, we examine top-ranked novel drug–disease pairs involving existing drugs and diseases on Gdataset for biological plausibility. Two examples illustrate concordance with external evidence. Leuprolide, a peptide-based GnRH receptor superagonist approved for palliative treatment of prostate cancer, uterine leiomyomata, endometriosis, and central precocious puberty, receives a high association score for premenopausal hormone receptor-positive (HR+) breast cancer. We note that Leuprolide targets protein GNRH1, and a *GNRH1* missense variant, rs6185 (p.Trp16Ser, c.47G>C), is significantly associated with age at menopause ($P$-value = 3E-13) [57]. Existing phase 3 clinical trials are investigating the safety, efficacy, and pharmacokinetic behavior of Leuprolide injectable emulsion for premenopausal breast cancer patients [58]. This is consistent with current clinical guidance supporting ovarian function suppression (OFS) combined with endocrine therapy, and with long-term suppression of ovarian function trial/ tamoxifen and exemestane trial updates showing improved outcomes when OFS is added [57]. Additionally, DREAM-GNN highlights a new association between Calcitriol and osteogenic sarcoma (osteosarcoma, OS). Calcitriol is a biologically active metabolite of vitamin D3 (1,25-dihydroxyvitamin D), an agonist of Vitamin D Receptor (VDR). Clinical evidence demonstrates that many patients with bone tumors, including OS, exhibit vitamin D deficiency [59]. Correspondingly, VDR is present in human OS tissues and cell lines, providing a biological basis for targeted therapy [60, 61]. Further, preclinical studies confirm that calcitriol exerts dose-dependent antiproliferative effects in multiple OS cell lines, capable of inducing cell cycle arrest, apoptosis, and endoplasmic reticulum stress responses [61]. Mechanistically, VDR signaling converges with multiple anti-tumorigenic pathways [62, 63]. This evidence provides a strong mechanistic rationale for evaluating vitamin D-based therapeutics, including calcitriol, in OS. Both examples highlight the biological validity of novel drug–disease pairs prioritized by DREAM-GNN, indicating its practical utility in pharmacological studies.

By leveraging DREAM-GNN, biomedical researchers are equipped with a powerful and generalizable framework for uncovering novel drug–disease associations with high precision, even in the face of noisy, incomplete, or heterogeneous data, a common challenge in real-world biomedical research. The model’s integration of biochemical, genomic, and clinical knowledge through pretrained embeddings enables the discovery of nonobvious therapeutic connections that may be overlooked by traditional approaches. This capability not only facilitates hypothesis generation for experimental validation but also supports the repurposing of approved or shelved compounds for emerging diseases, rare disorders, or personalized treatment strategies. In doing so, DREAM-GNN advances the translational utility of computational discovery, offering a scalable tool to bridge data-driven insights with therapeutic innovation.

While DREAM-GNN establishes a flexible and effective framework, there are areas for future enhancement. First, the model currently integrates features from chemical structures, protein sequences, and disease descriptions. Its predictive power could be further enhanced by incorporating additional biomedical knowledge, such as gene expression profiles, protein–protein interaction networks, and drug side-effect data, to construct a more comprehensive and informative heterogeneous graph. Second, although the attention mechanism offers some level of interpretability, future work should focus on developing more advanced techniques to translate the model’s predictions into biologically verifiable hypotheses regarding drug mechanisms of action. Third, as biomedical datasets continue to grow, exploring graph sampling techniques and memory optimization strategies will be crucial to ensure the framework remains computationally efficient when scaling to web-scale knowledge graphs. Fourth, residual transductive exposure remains a limitation, and future work will explore fully inductive training, temporal evaluation splits, and feature-only retrieval pipelines to eliminate node visibility and strengthen causal interpretability. Finally, beyond computational validation, the ultimate utility of DREAM-GNN depends on its biological relevance. Therefore, future work should prioritize experimental and clinical validation of model predictions to confirm their practical value in real-world drug discovery and translational medicine settings.

**Key Points**

- DREAM-GNN (Dual-Route Embedding-Aware Model for Graph Neural Networks) integrates rich, multimodal features from diverse sources, such as Simplified Molecular Input Line Entry System strings, protein sequences, and disease descriptions, by leveraging specialized pretrained biomedical language models to generate informative initial embeddings for drugs and diseases.
- DREAM-GNN employs a dual-route architecture: a relation-aware topology encoder captures structural patterns from known drug–disease associations, while a feature-aware similarity encoder derives relational signals from drug–drug and disease–disease similarity graphs, enabling the integration of complementary information from interaction topology and feature space geometry.
- DREAM-GNN significantly outperforms state-of-the-art methods, including DRAGNN (2024), AdaDR (2024), and DFDRNN (2025), across three benchmark datasets (Gdataset, Cdataset, and LRSSL), achieving gains of $\sim $6–12 percentage points in area under the precision-recall curve. Furthermore, under a strict cold-start protocol with unseen drugs or diseases, DREAM-GNN consistently maintains superior performance over all competing methods.

## Supplementary Material

supplement_bbaf555

## Data Availability

The source code for DREAM-GNN is publicly available at: https://github.com/Ryan-Yanlong/DREAM-GNN.
