# Supplementary material for: Dual-route embedding-aware graph neural networks for drug repositioning
Source: Brief Bioinform. 2025 Oct 27;26(5):bbaf555. doi: 10.1093/bib/bbaf555 (PMC12554636; doi:10.1093/bib/bbaf555)
Supplement: supplement_bbaf555 [file supplement_bbaf555.pdf]

Supplementary Information for "DREAM-GNN: Dual-route embedding-aware graph neural1 networks for drug repositioning"

Performance comparisons using F1-score and recall

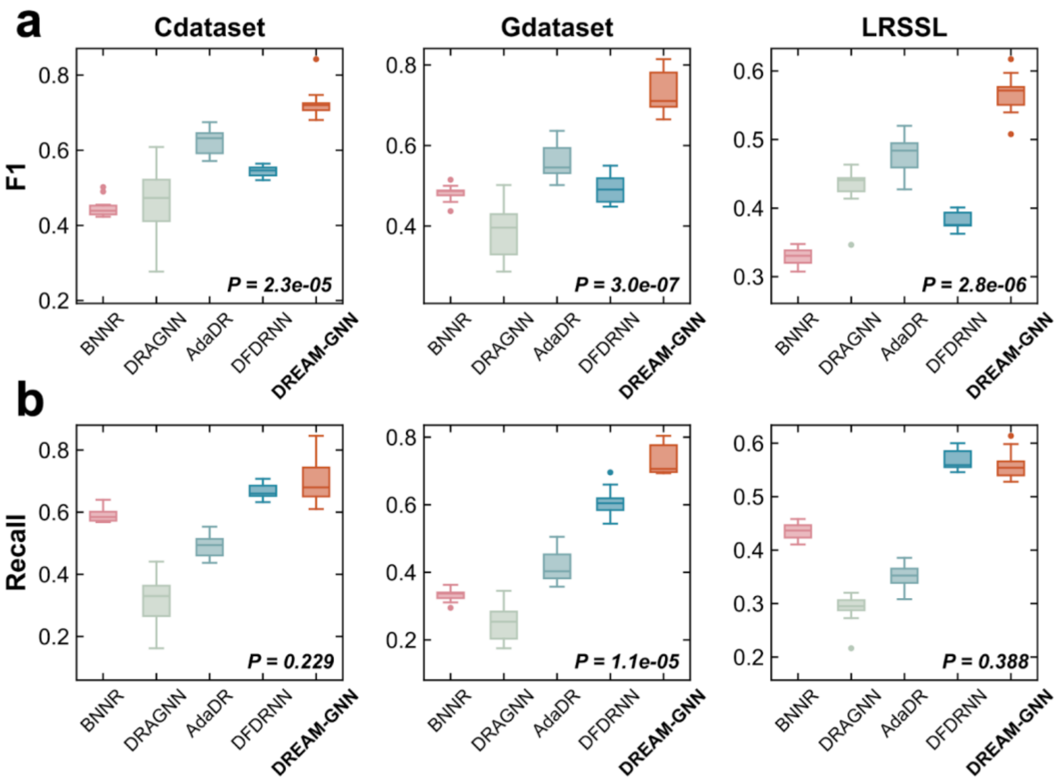

Fig. S1: Comparison of DREAM-GNN with existing methods across three datasets. DREAM-GNN outperforms BNNR, DRAGNN, AdaDR, and DFDRNN on both F1-Score (**a**) and Recall (**b**) metrics across Cdataset, Gdataset, and LRSSL. Boxplots reflect performance distributions over 10-fold cross-validation. *P*-values indicate statistical significance from paired t-tests against the strongest baseline for each dataset.
